# Systemic immune inflammation index and neutrophil-to-lymphocyte ratio as predictors of neonatal sepsis in low birth weight infants

**DOI:** 10.1186/s12887-026-07551-6

**Published:** 2026-08-24

**Authors:** Fiva Aprilia Kadi, Astrid Patricia Satyayu, Tetty Yuniati, Reni Ghrahani, Sri Endah Rahayuningsih, Dida Akhmad Gurnida

**Affiliations:** 1 Department of Child Health, Faculty of Medicine, Universitas Padjadjaran/Dr. Hasan Sadikin General Hospital, Bandung, West Java Indonesia; 2 Dr. Hasan Sadikin General Hospital, Pasteur 38, Bandung, 40161 West Java Indonesia

**Keywords:** Neonatal Sepsis, Low Birth Weight, Systemic Immune Inflammation Index, Neutrophil-to-Lymphocyte Ratio

## Abstract

**Background:**

The systemic immune inflammation index (SII) and neutrophil-to-lymphocyte ratio (NLR) are hematological parameters calculable from standard laboratory assessment. Despite extensive adult medicine implementation, neonatal applicability remains incompletely characterized. Early identification of sepsis in neonates remains challenging and sepsis is a significant global health problem associated with high morbidity and mortality, particularly among vulnerable low birth weight (LBW) infants. This study aimed to evaluate the predictive value of SII and NLR for neonatal sepsis in LBW infants.

**Methods:**

This observational analytic case–control study was conducted at Dr. Hasan Sadikin General Hospital, Bandung, between January 2024 and June 2025. A total of 290 LBW infants were enrolled in this study, comprising 145 infants with neonatal sepsis and 145 without sepsis. SII and NLR values were calculated from complete blood counts obtained at the initial evaluation within six hours after birth. Data were analyzed using receiver operating characteristic (ROC) curves to determine the area under the curve (AUC), optimal cut-off values, sensitivity, specificity and likelihood ratios (LR).

**Results:**

Both biomarkers were significantly associated with the subsequent development of culture-confirmed neonatal sepsis. Comparative predictive performance analysis revealed superior performance of SII (AUC: 0.778; 95%CI: 0.726–0.825) relative to NLR (AUC: 0.678; 95%CI: 0.620–0.731; *p* < 0.001). At an optimal cut-off value of ≤ 657,962.56, SII yielded a sensitivity of 57.9% and specificity of 88.3% with positive predictive value 83.2%, LR + 4.95 and LR − 0.48. The optimal NLR cut-off value of ≤ 3.1 showed a sensitivity of 59.3% and specificity of 73.1% with LR + of 2.20.

**Conclusions:**

Both SII and NLR are significant predictors of neonatal sepsis in LBW infants. SII demonstrates superior predictive performance and higher specificity compared with NLR.

## Background

The systemic immune inflammation index (SII) and neutrophil-to-lymphocyte ratio (NLR) are hematological indices that may serve as valuable predictors of neonatal sepsis [1, 2]. SII and NLR are rapid, inexpensive, and easily obtainable biomarkers derived from routine blood counts. Although these indices have been extensively studied in adult populations, their application in neonates remains limited. Global epidemiological data underscore persistent infection burden in neonates, with current estimates suggesting over one million cases annually [3]. Low birth weight (LBW) infants face heightened infection risk—approximately 1.4 to 1.5 times that of term infants—due to incomplete immune development characterized by deficient neutrophil bactericidal capacity and diminished complement activation [4].

While early diagnosis is imperative, it is frequently hampered by the non-specific nature of clinical presentations. Although blood culture is currently the diagnostic gold standard, its utility is constrained by modest diagnostic yield with a typical processing interval of 2–3 days, which often leads to delayed intervention or the indiscriminate use of empirical antibiotics [5]. Consequently, identifying rapid, cost-effective and widely accessible biomarkers is essential for optimizing prompt management and supporting antibiotic stewardship. This study aimed to evaluate whether admission SII and NLR, obtained from routine admission complete blood counts within the first six hours after birth, could predict the subsequent development of culture confirmed neonatal sepsis in LBW infants.

## Methods

### Study design and setting

This retrospective comparative analysis was a single-center study utilizing electronic medical records from Dr. Hasan Sadikin General Hospital (RSHS) in Bandung, Indonesia. The study was undertaken from January 2024 through June 2025.

### Study population

The study population comprised low birth weight infants born and admitted to Neonatal Intensive Care Unit (NICU) and Neonatal High Care Unit (NHCU) of Dr. Hasan Sadikin General Hospital, Bandung. Infants with positive blood culture results were included in the sepsis (case) group, whereas clinically non-septic infants served as controls. Infants with multiple congenital anomalies were excluded from the study.

### Sample size determination

The sample size was determined using receiver operating characteristic (ROC) curve analysis. Calculations were based on a two-sided significance level of 0.05 and a statistical power of 80%. Assuming an equal case-to-control ratio (1:1) and a prevalence of 50%, the minimum required sample size was 81 participants per group (total *n* = 162). The study population consisted of 290 LBW infants, stratified into 145 with microbiologically confirmed sepsis and 145 with uncomplicated clinical courses.

### Data collection

Eligible patients were first identified using International Classification of Diseases, Tenth Revision (ICD-10) diagnostic codes. Detailed demographic, clinical, and laboratory information was subsequently obtained through a comprehensive review of electronic medical records. Hematologic laboratory assessments were performed within the first six hours after birth using automated cell counters. SII was calculated as (neutrophil count × platelet count) ÷ lymphocyte count, while NLR was derived from neutrophil ÷ lymphocyte ratio. Sepsis was diagnosed based on clinical manifestations and laboratory results, including positive blood cultures. Missing data were handled using a complete-case approach. Only participants meeting all inclusion and exclusion criteria were included in the final analysis. A total of 459 infants were excluded because of incomplete laboratory or clinical data before application of the study eligibility criteria.

### Statistical analysis

Statistical analyses were conducted using IBM SPSS Statistics version 27.0 and MedCalc version 20. An independent t-test was used for normally distributed data, while the Mann-Whitney U test was applied for non-normally distributed data. Categorical variables were analyzed using the Chi-Square test. The predictive performance of SII and NLR was evaluated via ROC curve analysis, with the Youden Index utilized to determine the optimal cut-off values and Area Under the Curve (AUC). A p-value of ≤ 0.05 was considered statistically significant. Because universally accepted reference ranges for SII and NLR in LBW neonates are currently unavailable, no predefined normal or elevated values were used. Instead, optimal cut-off values were derived from receiver operating characteristic (ROC) curve analysis using the Youden Index and subsequently applied for predictive performance evaluation.

## Results

### Baseline characteristics

**Fig. 1:**
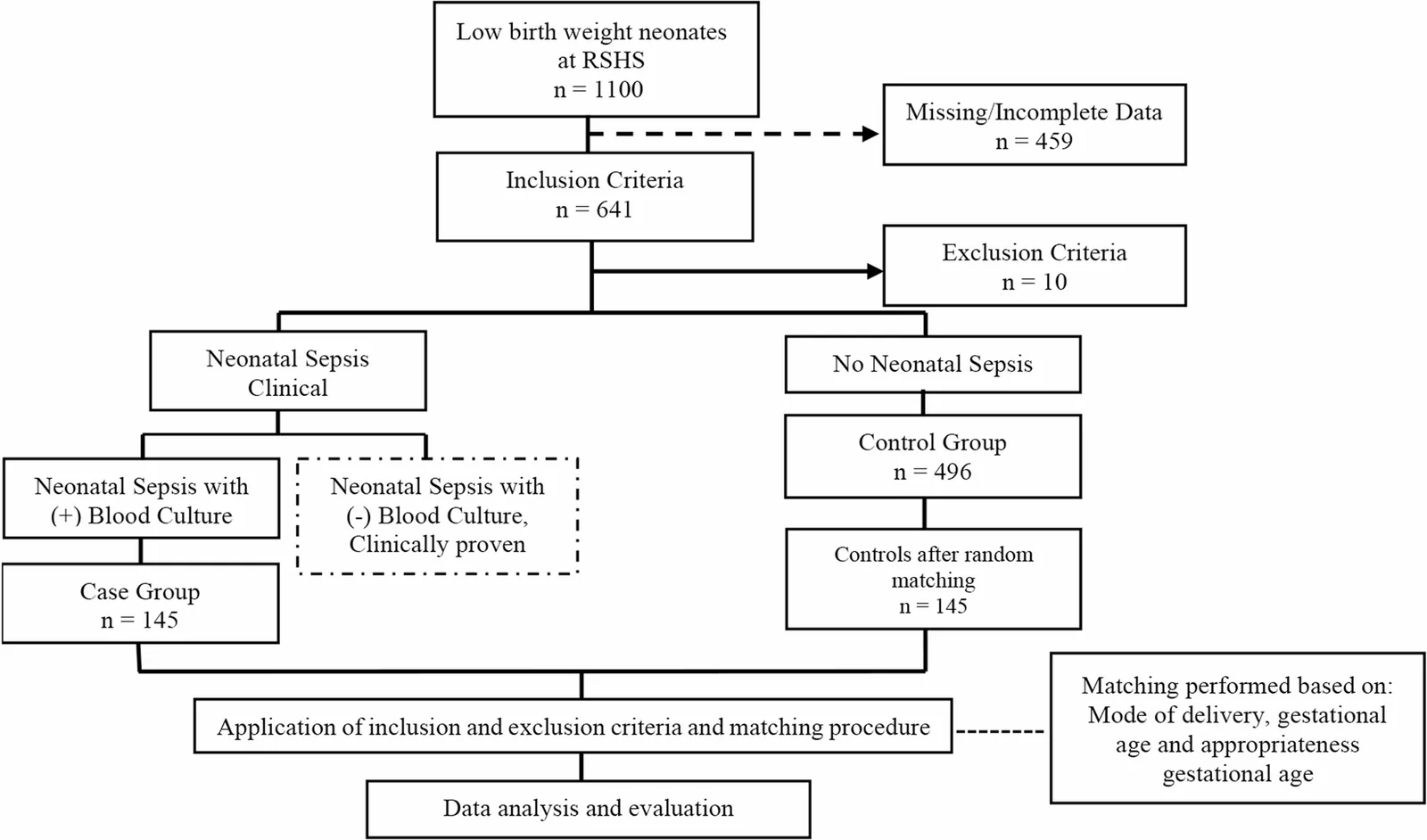
Flowchart of study subject selection

A total of 290 low birth weight (LBW) infants who met the inclusion and exclusion criteria were included in this study, comprising 145 infants with neonatal sepsis and 145 non-sepsis controls. Ten infants were excluded based on the predefined exclusion criteria, and the subject selection process is shown in Fig. 1.

Baseline characteristics were generally comparable among case and control groups. There were no statistically significant differences found in delivery method (*p* = 0.905), gestational age (*p* = 0.128), and gestational age appropriateness (*p* = 0.514). Significant differences were observed in sex, with male infants being more frequent in the sepsis group than in controls (57.9% vs. 40.7%; *p* = 0.003) and birth weight category differed significantly between groups (*p* < 0.001). Microbiological profile of the sepsis group was dominated by Late Onset Sepsis (LOS), with significant differences in blood culture results (*p* < 0.001) (Table 1).

**Table 1:** Baseline characteristics

Characteristics	Group		*P* value
	Sepsis	No Sepsis	
	*N* = 145	*N* = 145	
Sex			**0.003**
Male	84(57.9%)	59(40.7%)	
Female	61(42.1%)	86(59.3%)	
Method of Delivery			**0.905**
Spontaneous	61(42.1%)	60(41.4%)	
Sectio Caesarean	84(57.9%)	85(58.6%)	
Birth Weight			**< 0.001**
Low Birth Weight	81(55.9%)	139(95.9%)	
Very Low Birth Weight	48(33.1%)	5(3.4%)	
Extremely Low Birth Weight	16(11.0%)	1(0.7%)	
Gestational Age			**0.128**
Aterm	8(5.5%)	15(10.3%)	
Preterm	137(94.5%)	130(89.7%)	
Gestational Age Appropriateness			**0.514**
Small Gestational Age	13(9.0%)	10(6.9%)	
Appropriate Gestational Age	132(91.0%)	135(93.1%)	
Type of Sepsis			-
Early Onset Sepsis	10 (6.9%)	-	
Late Onset Sepsis	135 (93.1%)	-	
Blood Culture			**< 0.001**
Gram-Positive	10(6.9%)	0(0.0%)	
Gram-Negative	119(82.1%)	0(0.0%)	
Fungi	16(11.0%)	0(0.0%)	
None	0(0.0%)	145(100.0%)	
Thrombocyte			**< 0.001**
Mean±Standard deviation	215.862,07 ± 88.891,21	291.931,03 ± 78.824,85	
Neutrophils			**< 0.001**
Median	6.950,00	11.320,00	
* Range* (minimum–maximum)	1.640,00-35.480,00	2.490,00-30.090,00	
Lymphocytes			**< 0.001**
Median	2.680,00	2.960,00	
* Range* (minimum–maximum)	430,00-13.630,00	1.030,00-6.450,00	
SII			**< 0.001**
Median	553.979,59	1.073.723,92	
* Range* (minimum–maximum)	17.957,26 − 7,34E + 6	282.430,19 − 3,22E + 6	
NLR			**< 0.001**
Median	2.92	3.79	
* Range* (minimum–maximum)	0.82–14.86	1.39–10.61	

### Comparison of SII and NLR as factor predictor

Using the optimal cut-off derived from ROC analysis, SII ≤ 657,962.56 was more common among sepsis cases (57.9%) than controls (11.7%) (*p* < 0.001). Similarly, NLR ≤ 3.1 was more frequent in the case group (59.3%) than control groups (26.9%) (*p* < 0.001) (Table 2).

**Table 2:** Comparison between SII and NLR in LBW neonates with and without sepsis

Variable	Sepsis *N* = 145 (%)	Non-Sepsis *N* = 145 (%)	*P* value
SII Cutoff ≤ 657962,56 > 657962,56	84(57,9%) 61 (42,1%)	17(11,7%) 128(88,3%)	< 0,001
NLR Cutoff ≤ 3,1 > 3,1	86(59,3%) 59 (40,7%)	39(26,9%) 106 (73,1%)	< 0,001

As shown in Table 3, ROC analysis showed that the SII had good discriminatory ability for predicting neonatal sepsis (AUC 0.778; 95% CI, 0.726–0.825; *p* < 0.001), with high specificity at the optimal cut-off. The neutrophil-to-lymphocyte ratio (NLR) also demonstrated significant but lower discriminatory performance (AUC 0.678; 95% CI, 0.620–0.731; *p* < 0.001).

**Table 3:** Performance of SII and NLR as predictive factors

Parameter	SII	NLR	*P* value
AUC (95% CI)	0.778	0.678	< 0.001
Cut-off ROC	≤ 657962,56	≤ 3,1	
Sensitivity (%)	57.9	59.3	
Specificity (%)	88.3	73.1	
Positive Predictive Value (%)	83.2	68.8	
Negative Predictive Value (%)	67.7	64.2	
Likelihood Ratio +	4.95	2.20	
Likelihood Ratio -	0.48	0.56	

**Fig. 2:**
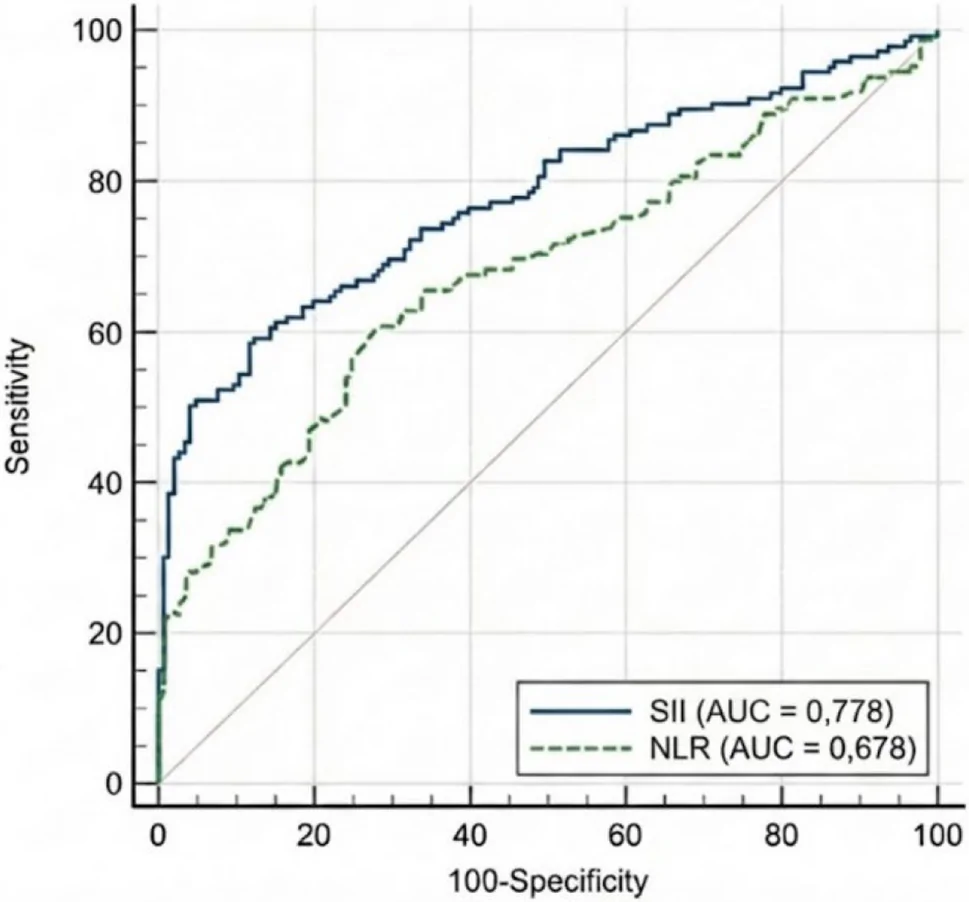
Comparison of AUC SII and NLR in Predicting Neonatal Sepsis in LBW

Figure 2 shows that the ROC curve for the SII has a larger area under the curve than that for the NLR, indicating better overall discrimination for neonatal sepsis in LBW infants.

## Discussion

Neonatal sepsis remains a major clinical challenge, particularly for early diagnosis in vulnerable newborns. Although global neonatal sepsis mortality has been reported to have decreased from 1990 to 2021 (average 0.75% per year), the burden remains substantial, with more than 550,000 neonatal deaths annually [6, 7]. Current literature indicates that approximately 84% of these sepsis related fatalities are preventable through a comprehensive understanding of risk profiles, followed by timely and targeted interventions [8]. 

This study revealed that male infants were more prevalent in the sepsis group, consistent with previous epidemiological studies identifying male sex as an independent risk factor for neonatal sepsis [9–11]. Proposed biological mechanisms include multifactorial genetic, immunologic, and hormonal factors; for example, dihydrotestosterone may exert immunosuppressive effects by reducing IL-4, IL-5, and interferon production, whereas estradiol may modulate the lipopolysaccharide-stimulated hypothalamic pituitary axis response [12]. Collectively, these findings suggest that LBW males warrant enhanced monitoring for early infection signals.

Birth weight showed a strong association with sepsis which in the case group included higher proportions of very low birth weight (VLBW, 33.1%) and extremely low birth weight (ELBW, 11.0%) infants compared with controls. This aligns with evidence that lower birth weight is a major risk factor for neonatal sepsis due to the physiological immaturity of the immune system in these infants [8, 10]. Because the study specifically focused on the heterogeneous spectrum of LBW infants, including LBW, VLBW, and ELBW neonates, birth weight was not included as a matching variable. Nevertheless, the imbalance in birth weight categories between groups may have influenced baseline hematologic parameters and should be considered when interpreting the findings.

Most cases in this study were LOS (93.1%), with microbiological profiles dominated by Gram-negative organisms (82.1%), followed by fungi (11.0%). Gram-negative infections are frequently associated with nosocomial acquisition and tend to provoke more severe systemic inflammation than Gram-positive infections [11]. Lipopolysaccharide released by Gram-negative organisms can induce hyperinflammation in LBW infants, who have limited bone marrow reserve that led to rapid peripheral consumption and bone marrow suppression, culminating in neutropenia and worse outcomes [13]. 

Statistical analysis showed that gestational age was not significantly different between groups, likely due to the relative homogeneity of the study population and the narrow range of gestational ages, with both groups consisting predominantly of preterm infants. In contrast, consistent with severe systemic infection in LBW infants, key hematologic components differed significantly between groups: platelet counts, neutrophils, and lymphocytes were all lower in the sepsis group. Thrombocytopenia is a sensitive marker in neonatal sepsis, and neutropenia in particular may indicate progression beyond the early inflammatory response into a more advanced septic phase, where immature marrow reserves are depleted and/or peripheral consumption is accelerated [14, 15].

A notable finding in this study is that both the SII and NLR were lower in the sepsis group than in controls. This contrasts with several studies in predominantly term populations reporting higher SII and NLR among septic neonates (e.g., Shanshan et al. in early-onset sepsis; Qigai et al. and Pantea et al. in late-onset sepsis) [4, 6, 16]. The discrepancy is plausibly explained by population differences, as LBW infants have an immature immune system and limited hematopoietic reserve, severe infection may manifest as cytopenias rather than “typical” neutrophilia driven inflammatory pattern. Therefore, low SII and NLR in this context should not be interpreted as absence of inflammation, but may reflect immune dysregulation, multisystem hematologic dysfunction and potentially poorer prognosis [4, 5]. Complete blood counts obtained at admission may help identify low birth weight infants at risk of subsequent neonatal sepsis. Their immature immune system and limited bone marrow reserve may be reflected in admission SII and NLR values before clinical infection develops, supporting the predictive value of these indices. According to a study by Ghrahani et al., preterm neonates with early onset sepsis and immature immune systems also exhibited lower leukocyte, neutrophil, and lymphocyte counts within the sepsis group [14]. This condition resulted in lower NLR values in the sepsis group compared to the non-sepsis group, aligning with the findings obtained in this study. The low SII cut-off in this study also aligns with the findings of Cakir et al., which focused on the VLBW population and identified that a low SII (cut-off ≤ 28.87, AUC 0.844) serves as an independent marker for predicting mortality [17]. 

Predictive performance analysis demonstrated that SII had better discriminative ability than NLR for predicting neonatal sepsis in LBW infants (AUC 0.778 vs. 0.678). The optimal SII cut-off (≤ 657,962.56) yielded moderate sensitivity (57.9%) but high specificity (88.3%) and a LR + of 4.95, supporting its potential clinical utility as an adjunctive rule-in biomarker when interpreted together with clinical findings and other laboratory investigations. In contrast, NLR at a cutoff of ≤ 3.1 showed modest performance (sensitivity 59.3%, specificity 73.1%, LR + 2.20). The lower AUC for NLR in this LBW cohort, compared with commonly reported ranges in neonatal sepsis (approximately 0.74–0.85) further supports the concept that LBW physiology and disease severity may shift hematologic patterns toward cytopenia [6]. Given their rapid availability from routine admission complete blood counts, SII and NLR may serve as useful adjunctive tools for early risk stratification of LBW infants. When interpreted alongside clinical assessment and other laboratory findings, these biomarkers may assist clinicians in identifying infants who warrant closer monitoring and timely evaluation for neonatal sepsis.

Several limitations should be considered when interpreting these findings. First, the retrospective case control design and single center setting may have introduced selection bias and limited the generalizability of the results. Second, important potential confounders, including maternal conditions and environmental exposures, were not evaluated, and the use of medical records may have affected data completeness and accuracy. Third, the predominance of preterm LBW infants, the small proportion of SGA infants, the high prevalence of late-onset sepsis, and the inclusion of only culture-confirmed sepsis cases may limit the applicability of the findings to other neonatal populations, including term infants and those with early-onset or culture-negative clinical sepsis. Finally, SII and NLR were derived from routine admission complete blood counts obtained within the first six hours after birth and were assessed using a single measurement. Therefore, the findings should be interpreted as reflecting the predictive value of early hematologic indices for subsequent neonatal sepsis rather than their diagnostic performance at the time of clinical suspicion. In addition, the proposed cut-off values were derived and evaluated within the same study cohort and have not undergone external validation; therefore, their applicability to other populations should be interpreted with caution. Larger prospective multicenter studies with balanced neonatal populations, serial biomarker measurements, and separate analyses of early- and late-onset sepsis are warranted to validate and refine gestational age specific predictive cut-off values. Furthermore, comparisons with established biomarkers such as CRP, procalcitonin, and IL-6 were not possible because these measurements were not consistently available in this retrospective cohort.

## Conclusion

Both SII and NLR are significant predictors of neonatal sepsis in LBW infants. SII demonstrates superior predictive performance and higher specificity compared with NLR.

## Data Availability

The datasets generated and/or analyzed during the current study are available from the corresponding author on reasonable request.

## Abbreviations

SII: Systemic immune inflammation index
NLR: Neutrophil-to-lymphocyte ratio
LBW: Low birth weight
VLBW: Very low birth weight
ELBW: Extrremely low birth weight
AUC: Area under the curve
CI: Confidence interval
ROC: Receiver operating characteristics
LOS: Late onset sepsis
